# The Utility of the Vasoactive-Inotropic Score and Its Nomogram in Guiding Postoperative Management in Heart Transplant Recipients

**DOI:** 10.3389/ti.2024.11354

**Published:** 2024-07-25

**Authors:** Tixiusi Xiong, Wai Yen Yim, Jiangyang Chi, Yixuan Wang, Hongwen Lan, Jing Zhang, Yongfeng Sun, Jiawei Shi, Si Chen, Nianguo Dong

**Affiliations:** Department of Cardiovascular Surgery, Union Hospital, Tongji Medical College, Huazhong University of Science and Technology, Wuhan, Hubei, China

**Keywords:** heart transplantation, survival, nomogram, vasoactive-inotropic score, early outcome

## Abstract

**Background:**

In the early postoperative stage after heart transplantation, there is a lack of predictive tools to guide postoperative management. Whether the vasoactive-inotropic score (VIS) can aid this prediction is not well illustrated.

**Methods:**

In total, 325 adult patients who underwent heart transplantation at our center between January 2015 and December 2018 were included. The maximum VIS (VIS_max_) within 24 h postoperatively was calculated. The Kaplan-Meier method was used for survival analysis. A logistic regression model was established to determine independent risk factors and to develop a nomogram for a composite severe adverse outcome combining early mortality and morbidity.

**Results:**

VIS_max_ was significantly associated with extensive early outcomes such as early death, renal injury, cardiac reoperation and mechanical circulatory support in a grade-dependent manner, and also predicted 90-day and 1-year survival (*p* < 0.05). A VIS-based nomogram for the severe adverse outcome was developed that included VIS_max_, preoperative advanced heart failure treatment, hemoglobin and serum creatinine. The nomogram was well calibrated (Hosmer-Lemeshow *p* = 0.424) with moderate to strong discrimination (C-index = 0.745) and good clinical utility.

**Conclusion:**

VIS_max_ is a valuable prognostic index in heart transplantation. In the early post-transplant stage, this VIS-based nomogram can easily aid intensive care clinicians in inferring recipient status and guiding postoperative management.

## Graphical Abstract

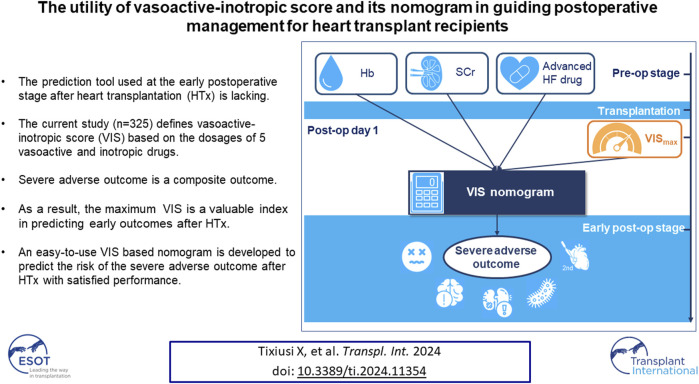

## Introduction

Heart transplantation is currently the final treatment for end-stage heart failure [1]. Developments in surgical technique and perioperative management have led to a significant decrease in post-transplant mortality [2]. However, 30-day mortality has remained unchanged at approximately 7% over the past decade [3]. Post-transplant morbidities are common and consistently worsen early recovery and long-term survival [2, 4, 5]. Thus, it is important to predict early mortality and morbidity in heart transplant recipients. While many models have been developed to predict the outcome of heart transplantation [6–8], only a few of them have been established for early outcomes in the hospital or within 90 days after transplant [9–11].

Compared with the preoperative prediction, which the majority of models perform to aid clinicians in making transplant decisions for a specific patient, the prediction in the early postoperative stage is also important. First, there is more information related to the transplant procedure and early postoperative recovery that can be used to improve outcome prediction in the early post-transplant stage than in the preoperative stage [6]. Second, a prediction model in the early postoperative stage can be utilized by intensive care unit (ICU) clinicians to infer the early recovery status of the recipient and guide subsequent management [12]. Nevertheless, relevant studies in heart transplantation are limited and a prediction tool early after transplantation is warranted.

The vasoactive-inotropic score (VIS) is a weighted sum of the doses of common vasoconstrictors and inotropes and is calculated during the first postoperative day or two [13]. It is considered a prognostic index of short-term outcomes in cardiac surgery patients [12, 14]. A VIS greater than 10 within the first 24 h post-transplant has been proposed as a criterion for primary graft dysfunction (PGD) by the consensus of the International Society of Heart and Lung Transplantation (ISHLT) [15]. Since PGD remains the leading cause of early mortality [16], the VIS index is thus expected to be useful in the outcome prediction of heart transplantation. However, the independent role of the VIS index in predicting outcomes after adult heart transplantation has not been adequately studied. The VIS index has been previously reported to be associated with early morbidities in adult and pediatric heart transplantation cohorts of small sample size [17, 18], but its relationship with mortality in different time scales was ambiguous [18]. Based on the above facts, we hypothesize that the VIS index can be used to develop an effective prediction model in the early postoperative stage for subsequent early outcomes after heart transplantation. Thus, we aim to explore the clinical value of VIS in predicting post-transplant outcomes and to construct an easy-to-use VIS-based nomogram for an early composite outcome in our heart transplant cohort that can be used by ICU clinicians to guide postoperative management of recipients.

## Methods

### Study Population

We included all adult patients who underwent orthotopic heart transplantation at our center between 1 January 2015 and 31 December 2018. Patients were excluded for: (1) Re-transplantation or multi-organ transplantation; (2) Immediate death within the first postoperative day; (3) Extreme body weight (<40 kg or >130 kg); (4) Lack of sufficient data on vasoactive-inotropic agents. After exclusion, 325 patients qualified for further analyses (Supplementary Figure S1). The donor hearts were all procured from voluntary donations after brain death and allocated using the China Organ Transplant Response System. The organs of executed prisoners were not used. Our research work conformed to the Declarations of Helsinki and Istanbul, and was approved by the Institutional Review Boards of Tongji Medical College. The requirement for patient consent was waived because the study’s nature was retrospective.

### Data Collection

We acquired patient data from the electronic medical record system. Among them, advanced heart failure treatment was defined as the preoperative administration of levosimendan or a recombinant human brain natriuretic peptide. The VIS was calculated using the formula modified from the inotrope score formula in the PGD consensus definition [16]: VIS = dopamine + dobutamine + 15 × milrinone + 100 × epinephrine + 100 × norepinephrine. Each item denotes the quotient of the drug dose (μg/min) divided by body weight (kg). Within the first 24 postoperative hours, the VIS at each hour was calculated and the maximum VIS (VIS_max_) [12] was obtained. Survival information was obtained through follow-up with the recipients and consultation with the related responsible doctors.

### Outcome Definitions

The primary outcome was the severe adverse outcome, a composite of early outcomes including early death, neurological complications, renal injury, septic shock and cardiac reoperation, which are commonly studied in cardiac patients [5, 12, 13]. The development of at least one of the above early outcomes was defined as the severe adverse outcome. Secondary outcomes were 90-day, 1-year and 6-year survival.

Early death was defined as in-hospital death or out-of-hospital death within 30 days of discharge [13]. Other complications all occurred in the hospital. Neurological complications were defined as the combination of stroke, as demonstrated by new cerebral deficits on radiological imaging, and seizure episodes requiring intervention. Renal injury was defined as newly initiated continuous renal replacement therapy (CRRT). Septic shock was defined as hypotension or hypoperfusion status with an infectious etiology. Cardiac reoperation was defined as a second thoracotomy after the initial transplantation.

### Statistical Analysis

Descriptive data were presented as “median (interquartile range)” or “mean (standard deviation)” for continuous variables, and as “number (percentage)” for categorical variables. Comparisons were performed by t-test or Mann-Whitney U-test for continuous data, and by Pearson χ^2^ test, continuity-adjusted χ^2^ test or Fisher’s exact probability test for categorical data. Survival curves were generated by the Kaplan-Meier method and their differences were examined using the Log rank test. Landmark analysis was undertaken for crossed survival curves. A logistic regression model was used to determine the independent risk factors for the severe adverse outcome. Clinical variables were selected according to clinical importance and the significance level in the univariate analysis of *p* < 0.1. All predictors were preoperative or intraoperative except VIS_max_. A correlation matrix was generated to assess all the continuous variables for collinearity. A forward stepwise method was used to screen variables for the multivariate model. The missing values for each variable were imputed using the multiple imputation method. A nomogram was constructed based on the multivariate logistic model. The regression coefficients in the model were used to derive linear predictors and allocate points in the nomogram.

The model’s performance was evaluated by calibration, discrimination and clinical utility. The calibration was assessed using a calibration plot and the Hosmer-Lemeshow test. The discrimination was assessed using the C-index or area under the curve (AUC) in the receiver operating characteristics (ROC) plot. The difference between the two AUCs was examined using DeLong’s method. The net reclassification index (NRI) and the integrated discrimination index (IDI) were calculated to determine whether the addition of a new index to the original model would improve the prediction. A decision curve analysis was performed to evaluate the clinical utility of the nomogram. Statistical analyses were conducted using SPSS v22.0 (SPSS, Chicago, IL, United States) and R v4.2.1 (The R Foundation for Statistical Computing, Vienna, Austria^1^ ). Figure plotting was completed using the same R software and GraphPad Prism v8.3.0 (GraphPad Software, San Diego, CA, United States). A *p*-value <0.05 was required for statistical significance.

## Results

### Demographic and Clinical Characteristics

The median age of our cohort was 50 years (IQR, 39.5–57 years), and the proportion of male patients was 78.46% (255/325). The median BMI was 22.81 kg/m^2 (IQR, 19.86–25.35 kg/m^2). After transplantation, the median VIS_max_ was 17.50 (12.92–24.90), and the rates for postoperative IABP and ECMO use were 37.23% (121/325) and 4.94% (16/325) respectively. Other demographic and clinical characteristics of the total cohort are summarized in Table 1. To explore the clinical value of VIS_max_, the cohort was divided into two groups according to its median. The high VIS_max_ group (VIS_max_ >17.5) had baseline variables that were overall comparable with the low VIS_max_ group (VIS_max_ ≤17.5) except for the ratios of lung disease history and preoperative dopamine usage (Table 1).

**TABLE 1:** Clinical characteristics and outcomes in different VIS_max_ groups.

Characteristics	Total cohort (n = 325)	VIS_max_		*p*-value
		Low (n = 163)	High (n = 162)	
Baseline				
Age (year)	50.00 (39.50–57.00)	51.00 (39.00–59.00)	49.00 (40.00–56.00)	0.315
Male patients	255 (78.46)	121 (74.23)	134 (82.72)	0.079
BMI (kg/m^2^)	22.81 (19.86–25.35)	22.83 (19.71–25.23)	22.77 (20.07–25.35)	0.947
Primary diagnosis				0.366
Non-ischemic cardiomyopathy	201 (61.85)	97 (59.51)	104 (64.20)	
Ischemic cardiomyopathy	67 (20.62)	33 (20.25)	34 (20.99)	
Valvular heart disease	40 (12.31)	21 (12.88)	19 (11.73)	
Others	17 (5.23)	12 (7.36)	5 (3.09)	
Diabetes mellitus	47 (14.46)	20 (12.27)	27 (16.67)	0.274
Lung disease	9 (2.77)	1 (0.61)	8 (4.94)	0.042
Kidney disease	20 (6.15)	8 (4.91)	12 (7.41)	0.367
Dopamine	184 (56.62)	82 (50.31)	102 (62.96)	0.025
Epinephrine	23 (7.08)	9 (5.52)	14 (8.64)	0.289
Advanced heart failure treatment	69 (21.23)	29 (17.79)	40 (24.69)	0.128
Hemoglobin (g/L)	136.00 (120.00–147.00)	136.00 (121.00–149.00)	137.00 (119.00–147.00)	0.885
Albumin (g/L)	39.45 (4.83)	39.60 (37.15–42.40)	39.00 (36.70–42.50)	0.406
Serum creatinine (μmol/L)	88.60 (71.30–105.30)	89.45 (72.48–107.63)	88.00 (71.05–105.15)	0.744
Total bilirubin (μmol/L)	21.20 (13.10–36.43)	19.90 (12.85–33.10)	23.40 (13.70–38.05)	0.123
Left ventricular ejection fraction (%)	26.00 (20.00–31.00)	26.00 (20.55–33.00)	25.55 (20.00–30.00)	0.306
Donor age (year)	35.00 (23.50–44.00)	35.00 (23.00–44.00)	35.50 (24.00–44.25)	0.507
Male donors	289 (89.20)	146 (90.12)	143 (88.27)	0.721
Donor BMI (kg/m^2^)	22.04 (20.76–23.88)	21.97 (20.76–24.22)	22.04 (20.76–23.63)	0.868
Cold ischemia time (min)	360.00 (300.00–404.00)	359.00 (289.25–411.00)	360.00 (300.00–400.00)	0.758
Postoperative				
VIS_max_	17.50 (12.92–24.90)	12.96 (10.26–15.38)	24.90 (20.51–31.63)	<0.001
IABP	121 (37.23)	29 (17.79)	92 (56.79)	<0.001
ECMO	16 (4.94)	2 (1.23)	14 (8.70)	0.002
Cardiac reoperation	14 (4.31)	3 (1.84)	11 (6.79)	0.031
CRRT	36 (11.08)	7 (4.29)	29 (17.90)	<0.001
Mechanical ventilation duration (h)	38.00 (24.00–59.48)	27.58 (21.40–41.50)	45.80 (33.83–89.91)	<0.001
ICU stay (h)	218.50 (168.00–281.00)	204.50 (158.75–253.50)	236.50 (180.50–321.75)	0.001
Respiratory complication	179 (55.08)	76 (46.63)	103 (63.58)	0.003
Neurological complication	16 (4.92)	6 (3.68)	10 (6.17)	0.319
Septic shock	9 (2.77)	2 (1.23)	7 (4.32)	0.173
Postoperative hospital stay (d)	31.00 (24.00–42.00)	29.00 (24.00–37.00)	34.00 (24.00–48.00)	0.005
Early death	32 (9.85)	10 (6.13)	22 (13.58)	0.026
Severe adverse outcome	63 (19.4)	21 (12.9)	42 (25.9)	0.003

Note: BMI, body mass index; VIS_max_, maximal vasoactive-inotropic score; IABP, Intra-aortic balloon pump; ECMO, extracorporeal membrane oxygenation; CRRT, continuous renal replacement therapy; ICU, intensive care unit.

### VIS_max_ and Post-Transplant Survival of 90-Day to 6-Year

**FIGURE 1:**
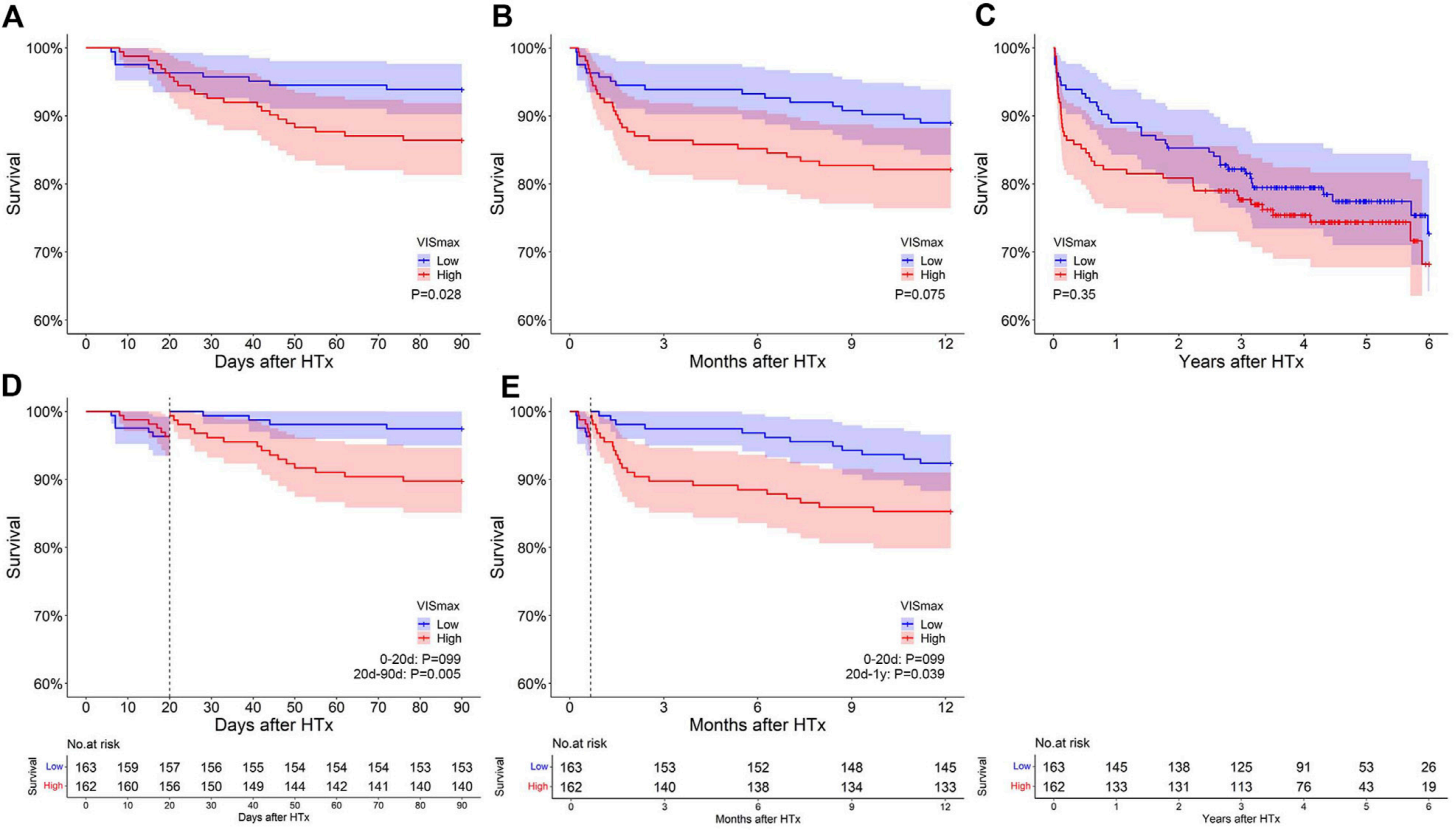
The impact of VIS_max_ on survival after heart transplantation at different follow-up periods. **(A–C)**: Original survival curves within 90 days, 1 year and 6 years after transplantation. **(D–E)**: Landmark analysis within 90 days and 1 year after transplantation.

The survival curves of the two VIS_max_ groups intersected at approximately day 20 within a 90-day and 1-year follow-up (Figures 1A, B). In the landmark analysis, no significant survival difference was observed before the intersection, while the survival of the low VIS_max_ group was evidently higher than that of the high VIS_max_ group after the intersection within a 90-day (*p* = 0.005) and 1-year follow-up (*p* = 0.039) (Figures 1D, E). Subsequently within a 6-year follow-up, the intersection became negligible and the survival difference between groups became not significant (Figure 1C). These results show that VIS_max_ is useful in predicting post-transplant survival in the short term rather than the long term.

### VIS_max_ Predicts Early Post-Transplant Mortality and Morbidity

**FIGURE 2:**
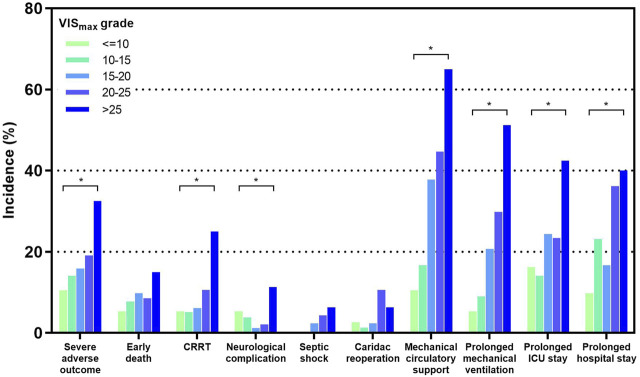
The incidences of early outcomes after heart transplantation in different VIS_max_ grade groups. *: *p* < 0.05 for multiple categorical comparisons. More details are presented in the Supplementary Material.

High VIS_max_ was significantly associated with various early post-transplant outcomes such as intra-aortic balloon pump (IABP), extracorporeal membrane oxygenation (ECMO), cardiac reoperation, secondary intubation, CRRT, respiratory system syndrome, early death, prolonged duration of mechanical ventilation, ICU stay and hospital stay (Table 1). We further divided our cohort into 5 groups with different VIS_max_ grades. Grades 1 to 5 corresponded to a VIS_max_ of: <=10, 10–15, 15–20, 20–25 and >25 respectively [13]. Significant increasing trends along with VIS_max_ grade existed in the rates of CRRT, mechanical circulatory support (IABP or ECMO), prolonged mechanical ventilation, ICU stay and hospital stay (*p* < 0.05), while a tendency for this trend existed for other outcomes such as early death, septic shock and cardiac reoperation (*p* > 0.05) (Figure 2). The above results show that VIS_max_ is associated with extensive early outcomes in a grade-dependent manner, indicating its predictive ability for an early composite outcome. The severe adverse outcome occurred in 19.4% of our patients and was also significantly associated with VIS_max_ in a grade-dependent manner (*p* < 0.05) (Figure 2).

### Establishment of a VIS-Based Predictive Model

For model establishment, a set of candidate variables included common preoperative variables such as recipient age, sex, BMI, diagnosis, and donor age, sex, BMI and cold ischemia time; intraoperative variables such as CPB duration and operation length; and VIS_max_ (Details are in Supplementary Table S1). The univariate logistic regression analyses were conducted to determine whether each candidate variable had a potential association with the severe adverse outcome (Supplementary Table S1). Forward stepwise selection in multivariate logistic modeling identified the following 4 variables independently related to the severe adverse outcome: VIS_max_ (OR: 1.055; 95%CI: 1.027–1.084; *p* < 0.001), hemoglobin (OR: 0.981; 95%CI: 0.967–0.996; *p* = 0.013), serum creatinine (OR: 1.012; 95%CI: 1.005–1.019; *p* = 0.001) and advanced heart failure treatment (OR: 2.499; 95%CI: 1.265–4.939; *p* = 0.008) (Table 2). This model established from the complete variable set was called the “complete model”. Next, by excluding VIS_max_ from the variable set of the complete model, a simplified set was generated and used to construct a control model. Similarly, in multivariate modeling, we identified 3 independent variables for the same outcome: hemoglobin (OR: 0.982; 95%CI: 0.968–0.997; *p* = 0.015), serum creatinine (OR: 1.012; 95%CI: 1.005–1.019; *p* = 0.001), advanced heart failure treatment (OR: 2.318; 95%CI: 1.208–4.448; *p* = 0.011) (Supplementary Table S2).

**TABLE 2:** Multivariate logistic model predicting severe adverse outcomes after heart transplantation.

Variables	β	Odds ratio (95% CI)	*p*-value
VIS_max_	0.054	1.055 (1.027–1.084)	<0.001
Hemoglobin (g/L)	−0.019	0.981 (0.967–0.996)	0.013
Serum creatinine (μmol/L)	0.012	1.012 (1.005–1.019)	0.001
Advanced heart failure treatment	0.916	2.499 (1.265–4.939)	0.008

Note: VIS_max_, maximum vasoactive-inotropic score.

### VIS-Based Nomogram and Its Performance

**FIGURE 3:**
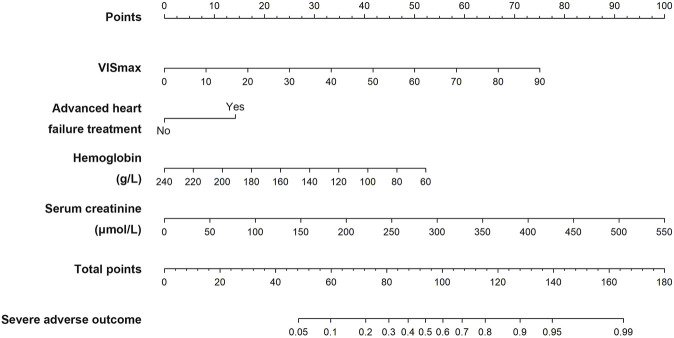
The VIS-based nomogram predicting the severe adverse outcome after heart transplantation.

The VIS-based nomogram for the severe adverse outcome in heart transplant recipients is shown in Figure 3. The points for each variable were summed up to generate a total score. A higher total score was related to a higher risk of the severe adverse outcome after heart transplantation. For example, a patient with a VIS_max_ of 7.62, hemoglobin of 96 g/L, serum creatinine of 70.7 μmol/L and no advanced heart failure treatment, would have 61.5 points (6.5 points for VIS_max_, 42 points for hemoglobin, 13 points for serum creatinine and 0 points for advanced heart failure treatment), for a predicted risk of the severe adverse outcome of 10.7%.

**FIGURE 4:**
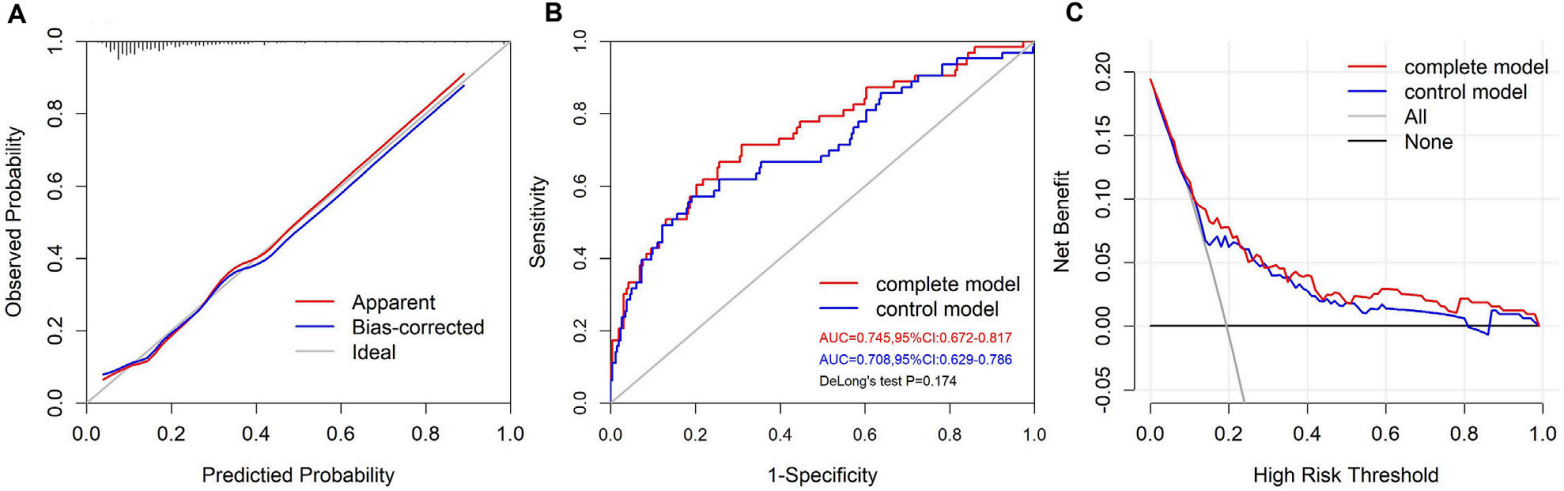
The performance of the VIS-based nomogram. **(A)** The calibration plot of the VIS-based nomogram. **(B)** The ROC curves of the VIS-based and control models. **(C)** The decision curves of the VIS-based and control models.

The calibration curve of the VIS-based nomogram was near the diagonal line (Figure 4A). The Hosmer-Lemeshow test yielded a χ^2^ of 8.094 (*p* = 0.424). There was a good agreement between the predicted and observed probabilities. The C-index was 0.745 (95%CI: 0.672–0.817) (Figure 4B), indicating moderate to strong discrimination. The prediction model after the removal of VIS_max_ is shown in Supplementary Table S2. The C-index for the control model was 0.708 (95%CI: 0.629–0.786), which was inferior to that of the complete model (Figure 4B). The addition of VIS_max_ to the control model resulted in a positive categorical NRI of 0.136 (*p* = 0.065), a significantly positive continuous NRI of 0.398 (*p* = 0.004), and a significantly positive IDI of 0.0485 (*p* = 0.006), suggesting a significant improvement in the risk classification ability of the model. The decision curve showed that when the selected interference threshold was >10%, using the VIS-based nomogram to predict the severe adverse outcome created more net clinical benefit than using a treat-all, a treat-none, and the control models (Figure 4C).

## Discussion

In this study, we explored the relationships of the VIS_max_ with early outcomes and survival at different time scales after heart transplantation. Based on the relevant preoperative and intraoperative variables and VIS_max_, a VIS-based nomogram was successfully developed with good performance in predicting the severe adverse outcome in heart transplant recipients.

The prognostic role of the VIS index on the early outcomes after heart transplantation in previous studies [17, 18] was confirmed in our study. Venema et al. divided 81 adult heart transplant recipients into three equal subgroups according to the mean VIS within 48 h postoperatively [18]. As a result, in-hospital outcomes such as ECMO, CRRT, and prolonged ICU and hospital stays were significantly associated with a high VIS index and the incidence of these outcomes was proportional to the VIS level. Our study confirmed the prognostic role of the VIS index on various early outcomes and more clearly depicted a similar grade-dependent manner in these associations using 5 subgroups and a graphic presentation. As for the impact of VIS on post-transplant survival, there were only a few relevant studies. A previous study discovered a significant association between the VIS index and 5-year mortality after adult heart transplantation but this association was inconsistent with different statistical methods and needed further verification [18]. In contrast, the present study found that VIS_max_ is a useful predictor of short-term survival (90 days, 1-year) rather than long-term survival, which enriches the clinical value of the VIS index.

The complete model incorporates four reasonable predictors. A higher VIS_max_ represents a higher dose of vasoactive and inotropic drugs administered postoperatively, suggesting a worse recovery status of patients in the early post-transplant stage. Thus, VIS_max_ may serve as a predictor of the severe adverse outcome. Taegtmeyer et al. demonstrated that pre-transplant anemia was significantly associated with 1-year mortality after heart transplantation [19], indicating that a lower level of preoperative hemoglobin may predict a worse post-transplant outcome, in line with our discovery. Reduced baseline kidney function may increase 30-day [20] and 1-year mortality [3] after heart transplantation, which supports our finding that an increase in preoperative serum creatinine is associated with a higher risk of the the severe adverse outcome. Advanced heart failure treatment in the present study includes the preoperative administration of levosimendan or recombinant human brain natriuretic peptide. These two drugs are used in our center to treat heart failure patients who cannot be relieved by conventional therapy. Therefore, preoperative advanced heart failure treatment is related to a subset of patients with worse baseline cardiac function, which may lead to a worse early outcome after transplantation.

Current prediction models [6–11] in heart transplantation are mostly established for preoperative prediction rather than early postoperative stage prediction, with only a few focusing on early in-hospital outcomes or within 90 days of transplantation. Singh et al. derived and validated a risk prediction model for in-hospital mortality after heart transplantation from a large registry [11], which calibrated well (Hosmer-Lemeshow *p* = 0.48) and had moderate discrimination (C-index = 0.68). The Index for Mortality Prediction After Cardiac Transplantation (IMPACT) is a model developed by Weiss et al. to predict 1-year survival after heart transplantation [8]. Figueredo et al. used IMPACT in their cohort of heart transplant recipients to predict in-hospital death with moderate to strong discrimination (C-index = 0.742) [21]. A more recent study by Nair et al. derived a prediction model named “GIMVECH” to determine the risk of post-transplant stroke [10] and obtained moderate discrimination (C-index = 0.65). However, the limited number of relevant articles reveals a lack of models for early outcomes after heart transplantation, particularly for prediction in the early postoperative stage. In the present study, we developed a VIS-based nomogram as a prediction tool in the early postoperative stage for the subsequent early composite outcome with good calibration and moderate to strong discrimination (C-index = 0.745). Despite the difference in the predicted outcome, the performance of this model is comparable to the performance of previous models.

Despite the association between PGD based on high VIS and increased early mortality after heart transplantation [16, 22], the independent role of VIS in predicting outcomes of heart transplantation has rarely been studied. Whether the use of VIS aids in predicting early post-transplant outcomes has not been verified. Meanwhile, post-transplant factors can have a significant impact on subsequent survival and can be used to improve the performance of predictive models [6]. In our study, we show the role of VIS_max_ in significantly improving the performance of the control model that incorporates only preoperative predictors, providing evidence for the importance of introducing post-transplant variables into outcome prediction for heart transplantation. The introduction of the VIS index makes the model capture a key feature of early postoperative recovery and consequently improves its performance. Meanwhile, this introduction also creates a good prediction tool in the early postoperative stage that can be utilized at the end of the first postoperative day to help ICU clinicians better identify high-risk recipients and formulate an individualized postoperative management plan.

There are several limitations to the present study. First, our cohort represents a cohort with high VIS and PGD rates, as evidenced by more than half of the recipients whose VIS_max_ was greater than 10 (actually 88.3% in the supplemental analysis) and 37.2% of the recipients using mechanical circulatory support postoperatively compared to the previously reported PGD rate (2.3%–28.2%) [15]. This fact may affect the generalizability of our nomogram to other centers. Second, the sample size of our cohort is relatively small compared to that of a large registry. A larger cohort is needed to confirm the prognostic role of VIS_max_. Third, no independent internal or external validation set is available to validate the model’s performance, which is needed in the future. Fourth, the VIS index has various forms in previous studies, but we only focused on the VIS_max_ within the first postoperative day based on 5 vasoactive-inotropic drugs. It remains to be studied whether other VIS indices can also predict the early post-transplant outcome.

## Conclusion

The VIS_max_ is a valuable prognostic index that predicts various early outcomes and short-term survival after heart transplantation and reflects the early postoperative recovery of recipients. In the early post-transplant stage, this VIS-based nomogram can be easily used by ICU clinicians for individualized prediction of subsequent early outcomes and to better guide the postoperative management of heart transplant recipients.

## Data Availability

The raw data supporting the conclusions of this article will be made available by the authors, without undue reservation.

## Footnotes

1
http://www.r-project.org

## Supplementary Material

SUPPLEMENTARY FIGURE S1 The flow chart of patient selection and follow-up. A total of 346 patients have undergone heart transplantation between 1 January 2015 and 31 December 2018 in our center, 1 of them was excluded for re-transplantation or multiple organ transplantation, 2 were excluded for immediate death with the first postoperative day, 3 were excluded for extreme body weight (130 kg) and 15 were excluded for lack of vasoactive-inotropic data. Every patient was followed up until the occurrence of primary outcome or the 30th day after discharge (around 60th day after transplantation). After follow-up, 63 patients have developed primary outcome.

## Abbreviations

AUC, Area under the curve; CI, Confidence interval; CRRT, Continuous renal replacement therapy; ECMO, Extracorporeal membrane oxygenation; IABP, Intra-aortic balloon pump; ICU, Intensive care unit; IDI, Integrated discrimination improvement; IQR, Interquartile range; NRI, Net reclassification index; OR, Odds ratio; PGD, Primary graft dysfunction; ROC, Recipient operating characteristics; VIS, Vasoactive-inotropic score.
