# Supplementary figures and images for: The Utility of the Vasoactive-Inotropic Score and Its Nomogram in Guiding Postoperative Management in Heart Transplant Recipients
Source: Transpl Int. 2024 Jul 25;37:11354. doi: 10.3389/ti.2024.11354 (PMC11306011; doi:10.3389/ti.2024.11354)

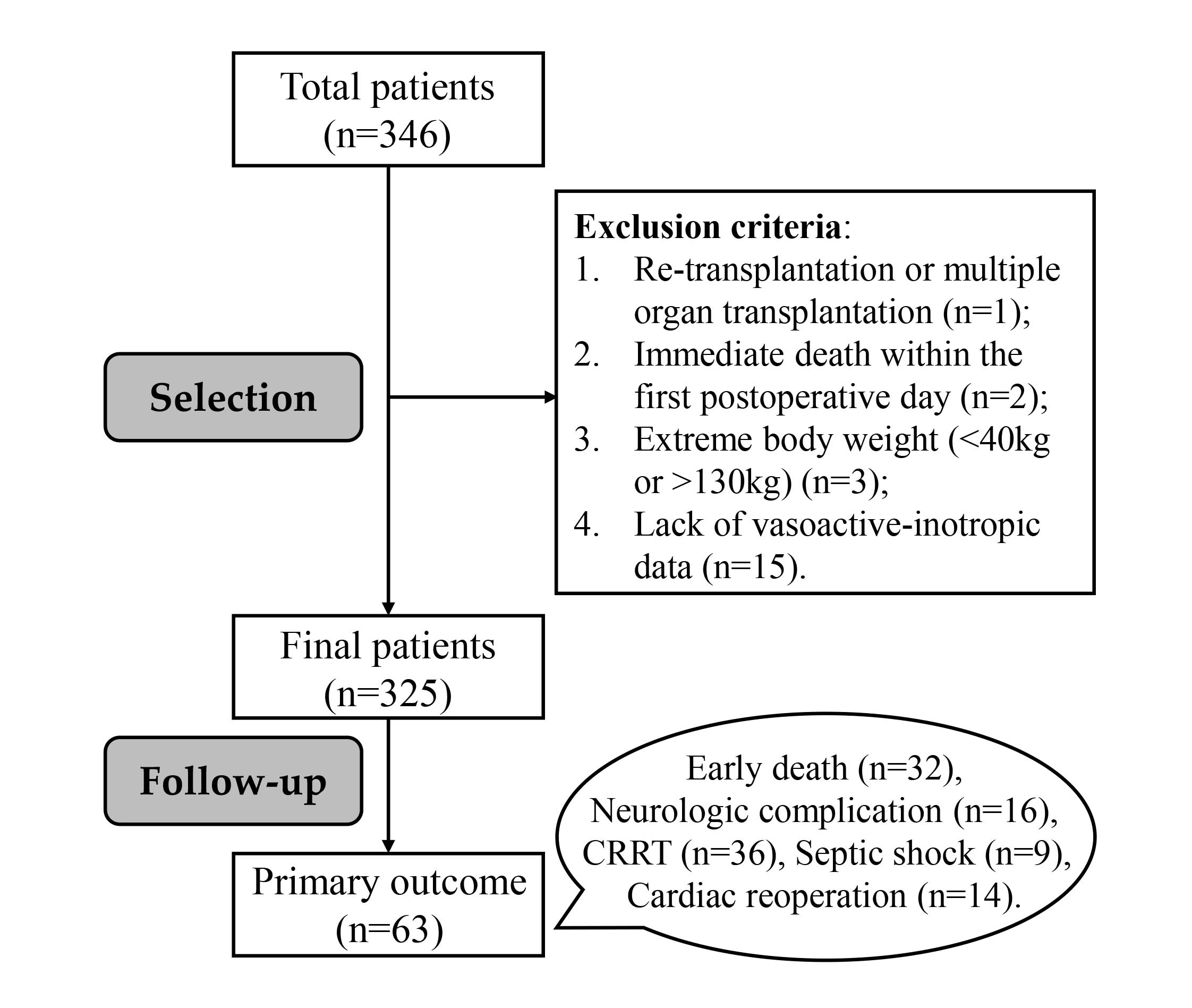

Supplement: Supplementary file 1 [file Image1.jpeg]
