# Supplementary material for: The Utility of the Vasoactive-Inotropic Score and Its Nomogram in Guiding Postoperative Management in Heart Transplant Recipients
Source: Transpl Int. 2024 Jul 25;37:11354. doi: 10.3389/ti.2024.11354 (PMC11306011; doi:10.3389/ti.2024.11354)
Supplement: Supplementary file 2 [file DataSheet1.docx]

**Supplemental Digital Content**

1. Supplementary tables

2. Supplementary material for Figure 2

3. Supplementary figure legends

**<1>Supplementary Tables**

| **Table S1 Univariate logistic models predicting severe adverse outcome after heart transplantation** | | | |
| --- | --- | --- | --- |
| **Variable** | **β** | **Odds ratio (95% CI)** | **P-value** |
| VIS_max_ | 0.049 | 1.050 (1.025-1.075) | <0.001 |
| Age (year) | 0.01 | 1.010 (0.987-1.033) | 0.409 |
| Male | 0.377 | 1.457 (0.774-2.744) | 0.243 |
| BMI (kg/m^2^) | 0.039 | 1.040 (0.966-1.119) | 0.296 |
| Primary diagnosis |  |  | 0.884 |
| Non-ischemic cardiomyopathy | 0.179 | 1.194 (0.328-4.358) | 0.786 |
| Ischemic cardiomyopathy | 0.116 | 1.123 (0.281-4.493) | 0.869 |
| Valvular heart disease | -0.194 | 0.824 (0.180-3.763) | 0.802 |
| Others | - | - | - |
| Diabetes mellitus | 0.684 | 1.983 (0.987-3.981) | 0.054 |
| Lung disease | 0.758 | 2.133 (0.519-8.774) | 0.294 |
| Kidney disease | 1.109 | 3.030 (1.183-7.765) | 0.021 |
| Dopamine | 0.794 | 2.212 (1.216-4.023) | 0.009 |
| Epinephrine | 0.815 | 2.259 (0.883-5.779) | 0.089 |
| Advanced heart failure treatment | 0.993 | 2.700 (1.476-4.938) | 0.001 |
| Hemoglobin (g/L) | -0.017 | 0.983 (0.970-0.997) | 0.014 |
| Albumin (g/L) | -0.078 | 0.925 (0.868-0.985) | 0.015 |
| Serum creatinine (μmol/L) | 0.013 | 1.013 (1.006-1.020) | <0.001 |
| Total bilirubin (μmol/L) | 0.01 | 1.010 (0.999-1.021) | 0.089 |
| Left ventricular  ejection fraction (%) | 0.007 | 1.007 (0.985-1.030) | 0.544 |
| CPB duration (min) | 0.014 | 1.014 (1.007-1.021) | <0.001 |
| Operation length (min) | 0.007 | 1.007 (1.004-1.011) | <0.001 |
| Donor age (year) | 0-0.001 | 1.000 (0.977-1.024) | 0.976 |
| Male donor | -1.028 | 0.358 (0.106-1.208) | 0.098 |
| Donor BMI (kg/m^2^) | -0.042 | 0.959 (0.871-1.056) | 0.393 |
| Cold ischemic time (min) | 0.001 | 1.001 (0.998-1.003) | 0.677 |
| Note: VIS_max_, maximal vasoactive-inotropic score; BMI, Body mass index; CPB, Cardiopulmonary bypass. | | | |

| **Table S2 Control multivariate logistic model predicting severe adverse outcome after heart transplantation** | | | |  |
| --- | --- | --- | --- | --- |
| **Variables** | **β** | **Odds ratio (95% CI)** | **P-value** | |
| Hemoglobin (g/L) | -0.018 | 0.982 (0.968-0.997) | 0.015 | |
| Serum creatinine (μmol/L) | 0.012 | 1.012 (1.005-1.019) | 0.001 | |
| Advanced heart failure treatment | 0.841 | 2.318 (1.208-4.448) | 0.011 | |
|  | | | |  |

**<2> Supplementary material for Figure 2**

**The detail of multiple comparison and pair comparison among 5 VIS_max_ grade groups.**

Multiple comparison among 5 groups was performed using Pearson χ^2^ test for severe adverse outcome, early death, CRRT, mechanical circulatory support, prolonged mechanical ventilation, prolonged ICU stay and prolonged hospital stay, and using Fisher’s exact test for neurological complication, septic shock and cardiac reoperation. The differences among groups were significant in the incidences of severe adverse outcome, CRRT, neurological complication, mechanical circulatory support, prolonged mechanical ventilation, prolonged ICU stay and prolonged hospital stay (P<0.05). For incidences of other outcomes, the differences were not significant (P>0.05). Comparisons between group pairs were conducted if the overall comparison among 5 groups reached a significant difference. Further, the comparison among the groups of lowest four VIS_max_ grades was conducted. If the consequent difference was not significant (P>0.05), then the comparisons between the fifth group and the lowest four groups were made. In this case, the comparison time was 5, and the significance probability was set to 0.01 (α_1_) according to Bonferroni method. Otherwise, the comparisons were made between every pair of the 5 groups. In this case, the comparison time was 10, and the significance probability was set to 0.005 (α_2_) according to Bonferroni method. For the incidence of severe adverse outcome, the lowest four groups had no significant difference from each other (P>0.05), the 5th group was significantly higher than the 1st and 2nd group (P<α_1_), and it had a critical difference from the 3rd group (P=0.013). For the incidence of renal injury requiring CRRT, the lowest four groups had no significant difference from each other (P>0.05), and the 5th group was significantly higher than the 2nd and 3rd group (P<α_1_). For the incidence of neurological complication, the lowest four groups had no significant difference from each other (P>0.05), and the 5th group was significantly higher than the 3rd group (P<α_1_). For the incidence of prolonged ICU stay, the lowest four groups had no significant difference from each other (P>0.05), and the 5th group was significantly higher than the 1st and 2nd group (P<α_1­_). For the incidence of postoperative mechanical circulatory support, all the pairs except the 4th and 5th group pair, the 3rd and 4th group pair, and the 1st and 2nd group pair, had a significant difference between the two component groups (P<α_2_). For the incidence of prolonged mechanical ventilation, the pairs except the pairs of the lowest three groups, the 3rd and 4th group pair, and the 4th and 5th group pair, had a significant difference between the two component groups (P<α_2_). For the incidence of prolonged hospital stay, only the 5th and 1st group pair, the 5th and 3nd group pair, and the 4th and 1st group pair had a significant difference (P<α_2_). For the incidence of early death, septic shock and cardiac reoperation, all the 5 groups had no significant difference from each other.

**<3> Supplementary figure legends**

**Figure S1 The flow chart of patient selection and follow-up**. A total of 346 patients have undergone heart transplantation between January 1, 2015 and December 31, 2018 in our center, 1 of them was excluded for re-transplantation or multiple organ transplantation, 2 were excluded for immediate death within the first postoperative day, 3 were excluded for extreme body weight (<40kg or >130kg) and 15 were excluded for lack of vasoactive-inotropic data. Every patient was followed up until the occurrence of primary outcome or the 30^th^ day after discharge (around 60th day after transplantation).
